# An Enzyme‐Responsive Self‐Immolative Recognition Marker for Manipulating Cell–Cell Interactions

**DOI:** 10.1002/advs.202402278

**Published:** 2024-07-02

**Authors:** Chad Plumet, Spyridon D. Katsakos, Mélissa Girard, Israa Al Jamal, Jonathan Clarhaut, Brigitte Renoux, Isabelle Opalinski, Sébastien Papot

**Affiliations:** ^1^ Equipe Labellisée Ligue Contre le Cancer UMR CNRS 7285 Institut de Chimie des Milieux et Matériaux de Poitiers (IC2MP) University of Poitiers 4 rue Michel‐Brunet, TSA 51106, Cedex 9 Poitiers 86073 France; ^2^ University Hospital of Poitiers 2 rue de la Milétrie Poitiers 86021 France

**Keywords:** bioorthogonal chemistry, chemical biology, metabolic glycoengineering, non‐covalent click chemistry, self‐immolative linker

## Abstract

The development of innovative strategies for cell membranes engineering is of prime interest to explore and manipulate cell–cell interactions. Herein, an enzyme‐sensitive recognition marker that can be introduced on cell surface via bioorthogonal chemistry is designed. Once functionalized in this fashion, the cells gain the ability to assemble with cell partners coated with the complementary marker through non‐covalent click chemistry. The artificial cell adhesion induces natural biological processes associated with cell proximity such as inhibiting cancer cell proliferation and migration. On the other hand, the enzymatic activation of the stimuli‐responsive marker triggers the disassembly of cells, thereby restoring the tumor cell proliferation and migration rates. Thus, the study shows that the ready‐to‐use complementary markers are valuable tools for controlling the formation and the breaking of bonds between cells, offering an easy way to investigate biological processes associated to cell proximity.

## Graphical abstract

An artificial enzyme responsive cell‐surface marker allowing to control the assembly of cancer and immune cells via non‐covalent click chemistry as well as their disassembly in response to an enzymatic stimulus is developed.

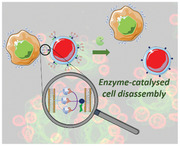

## 1 Introduction

The metabolic glycoengineering (MGE), which enables the installation of various bioorthogonal chemical functions on cell‐surface glycans, has emerged as a powerful tool to modify the outer membrane of living cells (for reviews see ref. [3]).^[^ ^1^, ^2^, ^3^ ^]^ Indeed, following MGE, the cell‐surface can be reshuffled by the introduction of a wide diversity of molecular systems via click chemistry, offering numerous potential biomedical applications including cell‐based therapies.^[^ ^4^ ^]^ In particular, artificial recognition markers have been incorporated into cell membranes through bioorthogonal ligation reactions with the aim to manipulate cellular interactions. Within this framework, different cell types that do not recognize naturally (e.g., tumor and immune cells) were coated with artificial surface markers and associated covalently using click chemistry^[^ ^5^, ^6^, ^7^, ^8^, ^9^ ^]^ as well as non‐covalently by the mean of complementary DNA sequences,^[^ ^10^ ^]^ aptamers^[^ ^11^ ^]^ or host/guest pairs.^[^ ^12^, ^13^ ^]^ Furthermore, some artificial markers were designed to allow both cell adhesion and dissociation in response to various stimuli like light,^[^ ^12^ ^]^ chemicals,^[^ ^6^ ^]^ or enzymes.^[^ ^10^ ^]^ When functionalized as such, cells can be considered as building blocks that can be assembled and separated through the formation and the breaking of chemical bonds, hence enabling the manipulation of cell–cell interactions in a stringently controlled manner with chemistry.

**Figure 1:**
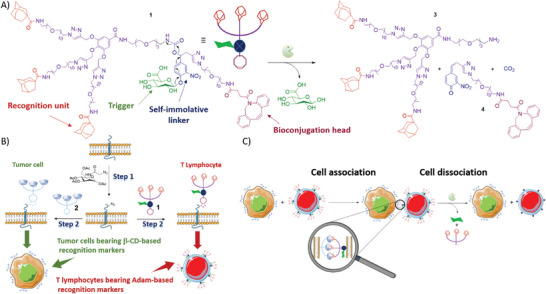
A) Structure and self‐immolation mechanism of the artificial recognition marker **1** in the presence of β‐glucuronidase; B) Principle of cell surface marker engineering with the complementary artificial markers **1** and **2**; C) Chemistry with cells: formation and breaking of bonds between cells.

Herein, we present the development of the artificial cell‐surface marker **1** that includes three adamantyl‐based recognition units,^[^ ^13^ ^]^ a glucuronide trigger,^[^ ^14^ ^]^ and a dibenzocyclooctyne (DBCO) bioconjugation head articulated around a self‐immolative linker^[^ ^15^ ^]^ (**Figure** **1**A). With this design, the enzyme‐sensitive molecular system **1** can be easily fixed on the membrane of cells previously treated by MGE, using the strain‐promoted azide‐alkyne cycloaddition (SPAAC, Figure 1B). Therefore, cells functionalized on a surface with the adamantyl marker **1** will acquire the ability to recognize cells coated with the complementary β‐cyclodextrins‐based (β‐CD) marker **2**,^[^ ^13^ ^]^ while these cells do not interact naturally (Figure 1C). On the other hand, in the presence of β‐glucuronidase (β‐Glu) the bond between cells will be disrupted by the enzyme‐catalyzed decomposition of **1**, thereby leading to cell disassembly (Figure 1A). Our study shows that the complementary surface markers **1** and **2** are ready‐to‐use chemical biology tools for manipulating cell–cell interactions and exploring their biological consequences. Indeed, these markers allow both the association of different cell types, such as immune and tumor cells, via non‐covalent click chemistry^[^ ^16^ ^]^ and the breaking up of their interactions in response to enzymatic stimulus. Furthermore, the unnatural intercellular binding affects cancer cell proliferation and motility while their ability to grow and migrate is restored by the rupture of cell adhesion. The versatility of our approach would allow the “on‐demand” assembly and disassembly of a wide range of cells in a stringently controlled fashion, hence providing a new avenue to manipulate cell–cell interactions and biological processes associated with cell proximity.

## 2 Results and Discussion

**Scheme 1:**
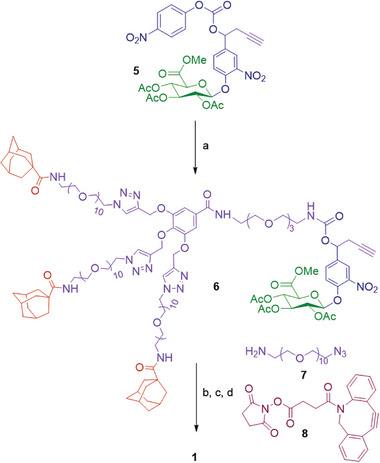
Synthesis of the artificial cell surface marker **1**. a) **3**, Et_3_N, DMF, rt, 2 h, **81%**; b) **7**, Cu(MeCN)_4_PF_6_, CH_2_Cl_2_, rt, 1.5 h; c) LiOH, H_2_O/MeOH, 0 °C, 30 min; d) **8**, Et_3_N, DMF, rt, 2 h, **25%** over three steps.

The artificial cell surface marker **1** was constructed employing a straightforward synthetic strategy from the two building blocks **3** ^[^ ^13^ ^]^ and **5** ^[^ ^15c^ ^]^ already described in the literature (**Scheme** **1**). First, these latter were coupled in the presence of Et_3_N to afford the carbamate **6** in 81% yield. The terminal alkyne of **6** reacted then with the commercially available *O*‐(2‐aminoethyl)‐*O’*‐(2‐azidoethyl)nonaethylene glycol **7** and Cu(MeCN)_4_PF_6_ to form the corresponding triazole that was engaged in the next step without further purification after the removal of the copper catalyst. The full deprotection of the glucuronide moiety was carried out using LiOH and the subsequent coupling with the *N*‐hydroxysuccinimide ester **8** gave the compound **1** in 25% yield over three steps.

**Figure 2:**
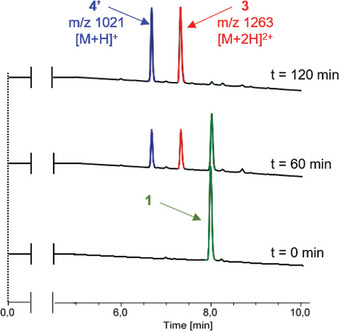
Enzymatic hydrolysis of **1** with *Escherichia coli* β‐glucuronidase in phosphate buffer (0.02 m, pH 7.2, 37 °C) monitored by HPLC/MS at t = 0, t = 60 min and t = 120 min. Retention times: **1** (8.01 min), **3** (7.33 min), **4′** (6.68 min).

We next investigated the decomposition mechanism of the artificial cell‐surface marker **1** in the presence of β‐Glu (Figure 1A). For this purpose, **1** was incubated with the enzyme in phosphate buffer (0.02 m, pH 7.2) at 37 °C, and the evolution of the mixture over the time monitored by HPLC/MS (**Figure** **2**).

The chromatograms showed the full disappearance of compound **1** within two hours under these conditions and the emergence of two new peaks with m/z 1263 [M + 2H]^2+^ and 1021 [M + H]^+^, which correspond respectively to the primary amine **3** and the *o*‐nitrophenol **4′** resulting from the addition of water on the quinone **4** (see the[Supplementary-material advs8837-supitem-0001]Supporting Information). This experiment confirmed that the enzyme‐catalyzed hydrolysis of the glycosidic bond of **1** was followed by the spontaneous decomposition of the linker, resulting in the separation of the recognition unit and the bioconjugation head (Figure 1A). Thus, such a cleavage process should lead to the disruption of interactions between cells associated via non‐covalent click chemistry by the mean of the complementary artificial markers **1** and **2** (Figure 1C).

**Figure 3:**
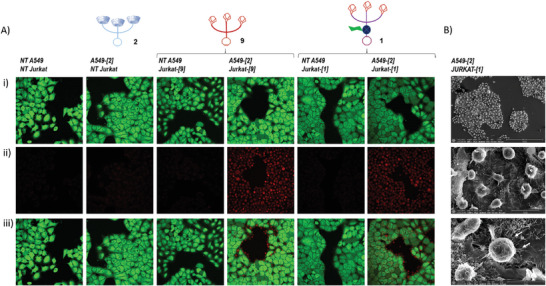
A) Confocal microscopy imaging of cellular recognition. Cells were incubated together for 10 min and washed with PBS prior to imaging: i) imaging of A549 adherent tumor cells (green); ii) imaging of Jurkat T cells (red); iii) merge. NT Jurkat: non treated Jurkat cells; NT A549: non treated A549 cells; Jurkat‐[**1**]: Jurkat cells coated with **1**; Jurkat‐[**9**]: Jurkat cells coated with **9**; A549‐[**2**]: A549 cells coated with **2**. B) Electron microscopy imaging of cellular recognition. Imaging of Jurkat T cell functionalized with the marker **1** (Jurkat‐[**1**]) on adherent A549 cells coated with the marker **2** (A549‐[**2**]) at three different magnifications; arrows show the appearance of filaments.

In order to verify this hypothesis, we first investigated the ability of **1** and **2** to trigger artificial cell recognition. For this purpose, human Jurkat T lymphocytes and A549 human cancer cells were incubated for three days with tetraacetylated *N*‐azidoacetyl‐*d*‐mannosamine (Ac_4_ManNAz) to functionalize their surface glycans with azides. Jurkat and A549 cells were then treated with **1** and **2**, respectively, for attaching the artificial recognition markers on their outer membrane via the SPAAC reaction. It is worth mentioning that such a membrane functionalization process did not affect cell viability (see the Supporting Information). Once modified, Jurkat T cells (Jurkat‐[**1**]) were seeded on A549 adherent cells (A549‐[**2**]) for ten minutes and the supernatant was removed. The adherent cells were next washed and fixed prior to analyze cell–cell interactions by confocal microscopy (**Figure** **3**A). As shown in Figure 3A, when both cells were not modified with the markers **1** and **2**, Jurkat T cells did not adhere to A549 cells, as testified by the absence of red fluorescence. In contrast, the bioorthogonal introduction of **1** and **2** on the membrane of Jurkat T and A549 cells triggered cell recognition. These results demonstrated that the complementarity of markers **1** and **2**, based on host‐guest interactions, induced the establishment of contacts between modified cells through non‐covalent click chemistry. The same experiments were also conducted with Jurkat T cells coated with the non‐cleavable marker **9** ^[^ ^13^ ^]^ used as a control (Jurkat‐[**9**]; for the full structure of **9**, see the Supporting Information). In this case, no significant differences were observed compared to the outcomes obtained with marker **1** indicating that the presence of the β‐glucuronidase‐responsive self‐immolative linker between the bioconjugation head and the three adamantyl‐based recognition units did not affect cell recognition.

The interactions between Jurkat‐[1] and A549‐[2] cells were also analyzed by electron microscopy (Figure 3B). These experiments confirmed the ability of engineered Jurkat T cells to recognize A549 tumor cells decorated with the β‐CD‐based marker 2. Furthermore, the pictures suggested that the interactions between the two cell types stimulated the remodeling of the actin cytoskeleton with the formation of filaments.^[^ ^17^ ^]^ This result showed that the non‐natural cellular adhesion induced by non‐covalent click chemistry can trigger some natural biological processes. Therefore, the complementary artificial markers 1 and 2 appeared as very useful chemical biology tools for exploring the biological consequences of cell proximity.

**Figure 4:**
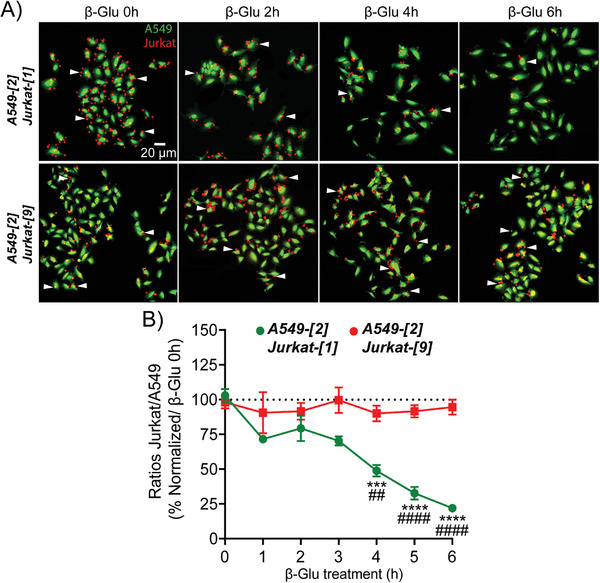
A) Confocal microscopy imaging of cellular recognition. A549‐[2] cancer cells (green) were associated with either Jurkat‐[1] or Jurkat‐[9] lymphocytes (red) and β‐Glu (133 units per mL) was incubated in the culture media. Pictures show cell association in the absence of β‐glucuronidase (β‐Glu 0 h) and 2, 4, and 6 h following the addition of the enzyme in the culture media. B) Quantification of the Jurkat‐[1] / A549‐[2] and Jurkat‐[9] / A549‐[2] ratio along the time after incubation of β‐Glu in the culture media. Values represent the mean ± SEM from three independent experiments. Statistical significance was determined by a Two‐Way ANOVA test with multiple comparisons followed by Tukey posttest (***p < 0001; ****p < 0,0001  = A549‐[2]/Jurkat‐[1] + β‐Glu 0 h vs A549‐[2]/Jurkat‐[1] + β‐Glu 4, 5, 6 h and ## : *p* < 0,01; ####*p* < 0,0001  = A549‐[2]/Jurkat‐[1] vs A549‐[2]/Jurkat‐[9]). β‐Glu: β‐glucuronidase.

We next investigated the enzyme‐catalyzed breaking of artificial cell–cell interactions. To this end, Jurkat‐[1] and A549‐[2] cells previously associated with non‐covalent click chemistry were incubated with β‐Glu, and cell adhesion was monitored by confocal microscopy (**Figure** **4**A). In these experiments, we analyzed the evolution of the Jurkat‐[1] / A549‐[2] ratio along the time for checking the ability of the enzyme to disrupt cell recognition (Figure 4B). The obtained results showed a significant decrease in the amount of Jurkat‐[1] associated with A549‐[2] cells. On the other hand, in the control experiments conducted with the non‐cleavable marker 9, such an effect was not observed since the Jurkat‐[9] / A549‐[2] ratio remained constant after the incubation of the β‐Glu in the culture media. The whole of these trials demonstrated that the artificial cell surface marker 1 enabled both the formation of intercellular bonds via host/guest interactions and the enzyme‐catalyzed cleavage of cell–cell contacts. This suggested that the stimuli‐responsive molecular system 1 could be used to manipulate biological processes related to intercellular adhesion by controlling “on‐demand” cell assembly and disassembly.

**Figure 5:**
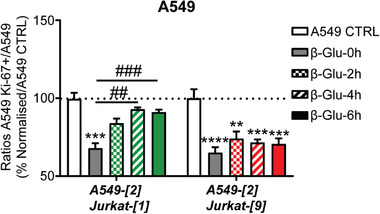
Quantifications of Ki‐67 positive A549‐[**2**] cancer cells associated with either Jurkat‐[**1**] or Jurkat‐[**9**] lymphocytes. The number of Ki‐67 positive A549‐[**2**] cancer cells was quantified along the time prior (β‐Glu‐0 h) and after addition of β‐Glu in the culture media (β‐Glu‐2 h, β‐Glu‐4 h, β‐Glu‐6 h). Values represent the mean ± SEM from three independent experiments. Statistical significance was determined by a Two‐Way ANOVA test with multiple comparisons followed by Tukey posttest (**p < 0,01; ***p < 0001, ****p < 0,0001  = A549 CTRL vs β‐Glu 0, 2, 4, 6 h and ## : *p* < 0,01; ### : *p* < 0001).

As proof of principle, we pursued our investigations by examining the impact of cell–cell interactions mediated by the complementary markers on cancer cell proliferation. Thus, A549‐[2] cancer cells were associated with either Jurkat‐[1] or Jurkat‐[9] lymphocytes, and cell proliferation was evaluated using Ki‐67 assay. As shown in **Figure** **5**, a decrease in the number of Ki‐67‐positive cancer cells was observed when Jurkat T cells adhered to the surface of A549 cells. These results indicated that cell proximity induced a reduction in the cancer cell proliferation rate. The same effect was noticed whether the artificial marker (1 or 9) attached to the surface of Jurkat lymphocytes, resulting in a 35% decrease in the proliferation rate (Figure 5, β‐Glu‐**0** **h)**.

We next incubated β‐Glu in the culture medium and continued to monitor cancer cell proliferation. When A549‐[**2**] cells were previously associated with Jurkat‐[**1**] lymphocytes, the initial proliferation rate of cancer cells was restored as a consequence of the enzyme‐catalyzed cell disassembly (Figure 5). In contrast, such a refurbishment was not observed with Jurkat‐[**9**] T cells that did not include a cleavable surface marker. Indeed, in this case A549 cell proliferation rate remained unchanged until 6 h after the addition of β‐Glu in the culture medium.

**Figure 6:**
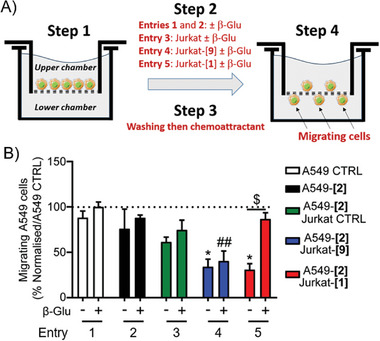
A) Schematic overview of cell migration assays. Step 1: incubation of A549 or A549‐[**2**] in the upper chamber; Step 2: incubation of Jurkat, Jurkat‐[**1**] or Jurkat‐[**9**] lymphocytes with A549‐[**2**] for 10 min; washing of the culture and incubation for 6 h in the presence or absence of β‐Glu; Step 3: washing of the culture medium and introduction of a chemoattractant (10% fetal bovine serum) in the lower chamber; Step 4: migrating cells were fixed and colored with crystal violet prior to be counted. B) Quantification of migrating A549 or A549‐[**2**] cancer cells. Values represent the mean ± SEM from four to five independent experiments. Statistical significance was determined by a Two‐Way ANOVA test with multiple comparisons followed by Tukey posttest (*p < 0,05 = A549 CTRL vs A549‐[**2**] + Jurkat‐[**9**]/Jurkat‐[**1**] without β‐Glu; ## : *p* < 0,01  = A549 CTRL vs A549‐[**2**] + Jurkat‐[**9**] with β‐Glu and $ : *p* < 0,05 = A549‐[**2**]/Jurkat‐[**1**] +/− β‐Glu.

We then used the complementary markers **1** and **2** to investigate the effects of artificial interactions on tumor cell motility. Cell migration plays indeed a fundamental role in the spread of cancer cells to form metastasis. To this end, we conducted migration assays employing Boyden chambers (**Figure** **6**A).^[^ ^18^ ^]^ In these experiments, either A549 or A549‐[**2**] cells were placed in the upper chamber and their ability to migrate was evaluated by counting the number of cells that moved towards the lower chamber after 48 h of incubation (see the Supporting Information).

As shown in Figure 6B, A549 and A549‐[**2**] cells exhibited comparable motility when incubated with or without β‐Glu (Entries 1 and 2). These results indicated that neither cell surface functionalization with the artificial marker **2** nor the presence of the enzyme in the culture medium affected significantly the migration capacity of cancer cells.

Similarly, co‐incubation of A549‐[**2**] cells with Jurkat lymphocytes did not modify significantly cancer cell motility (Entry 3). On the other hand, the association of A549‐[**2**] cells with Jurkat‐[**1**] or Jurkat‐[**9**] lymphocytes in the absence of β‐Glu limited cell migration as demonstrated by the decreased number of cancer cells detected in the lower chamber (Entries 4 and 5). However, the addition of β‐Glu in the culture medium restored the migration ability of A549‐[**2**] cells exclusively when they were associated with Jurkat‐[**1**] lymphocytes functionalized with the enzyme‐sensitive recognition marker **1** (Entries 4 and 5). Therefore, through the use of the self‐immolative molecular system **1**, these assays showed that cell contacts induced a decrease in cancer cell motility, while their ability to migrate can be retrieved via the breaking of cell–cell interactions.

Overall, these experiments confirmed that the stimuli‐responsive molecular system **1** allowed the manipulation of biological processes resulting from cellular proximity by controlling both cell assembly and disassembly.

## 3 Conclusion

In summary, we developed an artificial cell‐surface marker that can be attached via the SPAAC reaction on the outer membrane of living cells previously treated by MGE. Once modified on the surface, the cells can recognize other cell types functionalized with the complementary marker, through non‐covalent click chemistry. We showed that artificial cell–cell interactions can trigger natural biological processes promoted by cell proximity such as the remodeling of the actin cytoskeleton and the decrease in cancer cell proliferation or migration rate. Furthermore, we demonstrated that following enzymatic activation the stimuli‐responsive recognition marker can induce cell disassembly, hence canceling biological processes launched by cell proximity.

Since MGE can be applied to modify the surface of a wide range of cells, including eukaryote cells and bacteria, the ready‐to‐use complementary markers **1** and **2** could become versatile tools to explore the biological consequences of non‐natural cell contacts. The ability of the enzyme‐sensitive molecular system **1** to stimulate both cell assembly and disassembly could greatly facilitate the study of intercellular interactions, providing a better understanding of biological mechanisms associated with cell recognition. Additionally, **1** could be very useful for controlling the behavior of cellular networks with potential applications in tissue engineering. Thus, our complementary artificial markers will strengthen advantageously the current chemical biology toolbox to extend our knowledge of living systems.

## Conflict of Interest

The authors declare no conflict of interest.

## Supporting information

Supporting Information

## Data Availability

The data that support the findings of this study are available in the supplementary material of this article.
