# Supplementary material for: An Enzyme‐Responsive Self‐Immolative Recognition Marker for Manipulating Cell–Cell Interactions
Source: Adv Sci (Weinh). 2024 Jul 2;11(36):2402278. doi: 10.1002/advs.202402278 (PMC11423255; doi:10.1002/advs.202402278)
Supplement: Supplementary file 1 — Supporting Information [file ADVS-11-2402278-s001.docx]

**Supporting Information**

An enzyme-responsive self-immolative recognition marker for manipulating cell-cell interactions

Chad Plumet,^[a]^ Spyridon D. Katsakos,^[a]^ Mélissa Girard,^[a]^ Israa Al Jamal,^[a]^ Jonathan Clarhaut,^[a,b]^ Brigitte Renoux,^[a]^ Isabelle Opalinski^[a]^ and Sébastien Papot^[a]*^

*^[a]^ University of Poitiers, UMR-CNRS 7285, Institut de Chimie des Milieux et des Matériaux de Poitiers (IC2MP), 4 rue Michel Brunet, TSA 51106, 86073 Poitiers cedex 9, France.*

**E-mail:* [*sebastien.papot@univ-poitiers.fr*](mailto:sebastien.papot@univ-poitiers.fr)

*[b] University hospital of Poitiers, 2 rue de la Miléterie, 86021 Poitiers.*

***Table of Contents***

| **I. CHEMISTRY SECTION** | **S2** |
| --- | --- |
| **I.1. General experimental methods** | **S2** |
| **I.2. Synthetic overview of enzymatic-responsive self-immolative marker 1** | **S4** |
| **I.3. Synthetic procedures and characterization details with ^1^H NMR and ^13^C NMR NMR plots** | **S5** |
| **1.4 Evaluations of 1 in physiological conditions** | **S11** |
| **II. BIOLOGICAL SECTION** | **S13** |
| **II.1. Cell Culture** | **S13** |
| **II.2.Imaging of cell adhesion** | **S13** |
| **II.3. β-glucuronidase-catalyzed cell disassembly assays** | **S13** |
| **II.4. Cell proliferation assays** | **S15** |
| **II.5. Migration assays** | **S17** |
| **II.6. Cell viability assays** | **S18** |
| **III. REFERENCES** | **S19** |

**I. Chemistry Section**

**I.1. General experimental methods**

**Synthesis:** All reactions were performed under an argon atmosphere. Unless otherwise stated, solvents used were of HPLC quality. Chemicals were of analytical grade from commercial sources and were used without further purification. Reaction were monitored either by liquid chromatography, liquid chromatography coupled mass spectroscopy or using precoated silica gel TLC plates Macherey-Nagel ALUGRAM® SIL G/UV_254_. (0.2 mm silica gel 60). Spots were visualized under 254 nm UV light and/or by dipping the TLC plate into a solution of phosphomolybdic acid (3 g) in ethanol (100 mL) followed by heating with a heat gun. Automatic flash chromatographies were performed with a COMBIFLASH® RF 200I TELEDYNE ISCO instrument equipped with UV and ELSD detectors. Normal phase chromatographies were run using flash cartridges from Interchim®. Silica particles size (from 15 μm to 50 μm) and columns size (4 g to 280 g) were adapted according the difficulty of the purification and the quantity of crude product. Reverse phase chromatographies were run using flash cartridges HP C18 RediSep® GOLD 4 g, 15.5 g.or 30 g.

**Characterization: ^1^H and ^13^C NMR** spectra were respectively recorded at 500 MHz, 126 MHz on a Bruker 500 Avance NEO instrument, equipped with an ultrashielded magnet and a Prodigy cryoprobe. Chemical shifts (δ) are reported in parts per million (ppm) from low to high field and referenced to residual solvent peaks. Coupling constants (*J*) are reported in hertz (Hz). Standard abbreviations indicating multiplicity are used as follows: b = broad, s = singlet, d = doublet, t = triplet, q = quartet, qi = quintet, m = multiplet, dd = doublet of doublets. **High-resolution mass spectra** (HRMS) were performed on a LC-QTof MaXis Impact, Bruker by the mass spectrometry service of IC2MP (Poitiers, UMR 7285, platform Platina)

**Analysis:** **Analytical LC-MS** was performed on a Shimadzu LCMS-2020. A reverse-phase column chromatography MACHEREY-NAGEL NUCLEOSHELL® (150/4.6, RP18, 5 µm) at 40°C was used for chromatographic separation at a flow rate of 1 mL.min-1. The column effluent was introduced into the electrospray ionization source (ESI) of the mass spectrometer. Analyses were performed in positive and negative ion modes. The electrospray voltage was set at 4.5 kV. The capillary and heater temperatures were 250°C and 400°C respectively. The drying gas (nitrogen) and nebulizing gas (nitrogen) flow were set at 15 L.min^-1^ and 1.5 L.min^-1^ respectively. Analysis of data was performed with LabSolutions software. LC/MS experiments were performed using a linear gradient composed of A (0.1% formic acid in water) and B (0.1% formic acid in CH_3_CN) starting from 20% of B and reaching 73% of B within 10 min.

**Analytical RP-HPLC** was performed on DIONEX Ultimate 3000 with UV light set to 214 nm with MACHEREY-NAGEL Nucleoshell®(150/4.6, RP18, 5 μm) column in a thermostatically controlled oven at 30 °C. Spectra analysis was carried out with the software Chromeleon. Eluents were A (H_2_O + 0.2% TFA), B (MeCN) and solvent flow: 1.2 mL.min^-1^. Method: isocratic gradient A/B 80:20 for 1 minute, then linear gradient reaching A/B 0:100 within 10 minutes, then isocratic A/B 0:100 for 2 minutes and linear gradient toward A/B 80:20 within 2 minutes.

**The following compounds were synthesized according to literature procedures:**

**Scheme S1.** *References of literature procedures:* compounds **2**, **3** and **9**^1^ compound **5^2^**.

**I.2. Synthetic overview of enzyme-responsive self-immolative marker 1.**

Compound **1** was prepared according to the following strategy:

**Scheme S2.** *Reagents and conditions:* (a) Et_3_N, DMF, RT, 2 h, 81%; (b) **7**, Cu(MeCN)_4_PF_6_, DCM, RT, 1 h 30, not purified; (c) i) LiOH/H_2_O, MeOH, 0 °C, 30 min; ii) **8**, Et_3_N, DMF, RT, 2 h, 25% (three steps).

**I.3. Synthetic procedures and characterization details with ^1^H NMR and ^13^C NMR plots**

***Preparation of compound 6***

To a solution of triadamantyl **3** (52.6 mg, 0.0216 mmol, 1 equiv.) in dry DMF (2 mL) was added carbonate **5** (16.4 mg, 0.0238 mmol, 1.1 equiv.) and Et_3_N (15.1 µL, 0.108 mmol, 5 equiv.). The mixture was stirred at room temperature during 2 hours and the progress of the reaction was monitored by LC/MS. After completion of the starting material, the solvent was evaporated under reduced pressure and the crude material was purified by reverse phase chromatography (H_2_O (0.05% TFA)/MeCN, gradient elution 60:40 to 30:70) to isolate compound **6** (54 mg, 81%) as a colorless oil.

**^1^H NMR** (500 MHz, CD_2_Cl_2_) δ 7.98 (s, 2H, H_3_), 7.86 (s, 1H, H_3’_), 7.83 (d, ^4^*J* = 1.9, 1H, H_a_), 7.57-7.54 (m, 1H, H_b_), 7.33 (d, ^3^*J* = 8.6, 1H, H_c_), 7.28 (s, 2H, H_1_), 7.20 (s, 1H, H_21_), 6.12 (s, 3H, H_8-8’_), 5.84-5.82 (m, 1H, H_12_), 5.72 (t, ^3^*J* = 6.3, 1H, H_d_), 5.37 – 5.25 (m, H_glucuronide_, masked partially by CD_2_Cl_2_), 5.22 (s, 4H, H_2_), 5.15 (s, 2H, H_2’_), 4.56 (t, ^3^*J* = 5.0, 4H, H_4_), 4.50 (t, ^3^*J* = 5.2, 2H, H_4’_), 4.31 (dd, *J* = 9.2, 4.1, 1H, H_glucuronide_), 3.89 (t, ^3^*J* = 5.1, 4H, H_5_), 3.84 (t, ^3^*J* = 5.2, 2H, H_5’_), 3.69 (s, 3H, H_OMe_), 3.67 – 3.52 (m, 123H, H_PEG_), 3.51 (t, ^3^*J* = 5.2, 6H, H_PEG_), 3.48-3.43 (m, 2H, H_PEG_), 3.39 – 3.36 (m, 6H, H_7-7’_), 3.27-3.24 (m, 2H, H_13_), 2.77-2.67 (m, 2H, H_e_), 2.06 (s, 4H, H_OAc-f_), 2.03 (s, 6H, H_OAc_), 2.00 (s, 9H, H_10-10’_), 1.81 (d, *J* = 2.5, 18H, H_9-9’_), 1.75 – 1.67 (m, 18H, H_11-11’)_.

**^13^C NMR** (126 MHz, CD_2_Cl_2_) δ 178.1, 170.1, 169.7, 169.5, 167.2, 155.4, 152.5, 149.1, 144.3, 143.5, 141.1, 140.3, 136.2, 132.5, 130.7, 125.1, 125.0, 123.6, 119.0, 107.2, 99.7, 79.2, 72.7, 72.5, 71.8, 71.3, 71.3, 70.8, 70.8, 70.8, 70.7, 70.7, 70.6, 70.6, 70.4, 70.3, 70.2, 70.1, 69.7, 69.7, 69.1, 66.5, 63.1, 50.7, 40.8, 39.5, 39.3, 36.9, 28.7, 20.8, 20.7, 20.7.

**HRMS (ESI^+^)** *m/z* = 1559.2865 [M+2Na]^2+^ (calc. for C_147_H_233_N_15_Na_2_O_54_: 1559.2844 [M+2Na]^2+^), *m/z* = 1575.2605 [M+2K]^2+^ (calc. for C_147_H_233_N_15_K_2_O_54_: 1575.2616 [M+2K]^2+^)

^1^H NMR spectrum of **6**, 500 MHz, 298 K, CD_2_Cl_2_

^13^C NMR spectrum of **6**, 126 MHz, 298 K, CD_2_Cl_2_

***Preparation of compound 1***

Compound **6** (62.8 mg, 0.0204 mmol, 1 equiv.) and azido-PEG_10_-amine **7** (11.8 mg, 0.0225 mmol, 1.1 equiv.) were dissolved in dry DCM (2 mL). The mixture was degassed under argon atmosphere. Cu(MeCN)_4_PF_6_ (11.4 mg, 0.0306 mmol, 1.5 equiv) was added and the solution was allowed to stir for 2 hours at room temperature. The reaction was monitored by HPLC. Resin QuadraPure® IDA (200 mg) was added to the mixture which was allowed to stir for an additional hour. The resin was then filtered and washed with MeOH. The solvent was evaporated. The crude product was dissolved in MeOH (2 mL) and cooled to 0 °C. LiOH (7.6 mg, 0.180 mmol, 8.8 equiv.) in 1 mL of cooled water was slowly added and the mixture was stirred at 0 °C for 30 minutes. After completion, the solution was neutralized by adding Amberlyst IR-15 resin. After filtration and evaporation of the solvent, the new crude residue was dissolved in dry DMSO (2 mL) and Dibenzocyclooctyne-*N*-hydroxysuccinimidyl ester **8** (9.04 mg, 0.0224 mmol, 1.1 equiv.) followed by Et_3_N (8.5 µL, 0.0613 mmol, 3 equiv) were added. The reaction was stirred overnight at room temperature and monitored by HPLC and LC/MS. After total consumption, the solvent was evaporated under reduced pressure. The crude residue was purified by reverse phase chromatography (H_2_O (0.05% TFA)/MeCN, gradient elution 70:30 to 30:70) to isolate compound **1** (19 mg, 25% over 3 steps) as a colorless oil.

**^1^H NMR** (500 MHz, CD_2_Cl_2_) δ 8.01 (s, 2H, H_3_), 7.91 (s, 1H, H_3’_), 7.71 (s, 1H, H_f_), 7.65 (d, ^3^*J* = 7.4, 1H), 7.53 – 7.50 (m, 3H), 7.44 – 7.24 (m, 9H), 6.54 (bs, 1H, H_12_), 6.24 (bs, 3H, H_8-8’_), 6.03 – 5.97 (m, 1H, H_23”’_), 5.85 (m, 1H), 5.19 (s, 4H, H_2_), 5.15 (s, 2H, H_2’_), 5.13-5.10 (m, 2H), 5.04 (t, ^3^J=7.5 Hz, 1H, Η_d_), 4.56 (t, ^3^*J* = 4.6, 4H, H_4_), 4.51 (t, ^3^*J* = 4.9, 2H, H_4’_), 4.47 (t, ^3^*J* = 4.8, 2H, H_1”’_), 4.05 (m, 1H), 3.89 (t, ^3^*J* = 4.9, 4H, H_5_), 3.85 (t, ^3^*J* = 5.1, 2H, H_5’_), 3.78 (m, ^3^*J* = 4.8, 2H, H_2”’_), 3.67 (s, 1H), 3.65 – 3.41 (m, 170H), 3.38 (m, 6H, H_7-7’_), 3.29 – 3.26 (m, 3H), 3.23 – 3.15 (m, 2H), 2.80 – 2.67 (m, 1H, H_24”’ or 25”’_), 2.63 (s, 4H), 2.40 – 2.35 (m, 1H, H_24”’ or 25”’_), 2.19 – 2.13 (m, 1H, H_24”’ or 25”’_), 2,00 (s, 9H, H_10-10’_), 1.96-1.88 (m, 1H, H_24”’ or 25”’_), 1,81 (d, *J* = 2.5, 18H, H_9-9’_), 1.75 – 1.67 (m, 18H, H_11-11’_).

**^13^C NMR** (126 MHz, CD_2_Cl_2_) δ 178.6, 152.4, 151.8, 149.8, 148.8, 143.4, 140.2, 135.8, 132.7, 129.8, 129.0, 128.5, 128.3, 128.0, 127.3, 125.8, 125.2, 123.6, 122.7, 118.5, 114.8, 108.3, 107.3, 76.1, 75.0, 73.8, 73.2, 71.6, 70.7, 70.7, 70.6, 70.6, 70.5, 70.1, 70.0, 70.0, 69.6, 66.1, 62.9, 55.8, 54.3, 54.1, 53.8, 53.6, 53.4, 50.8, 41.2, 40.8, 40.7, 40.4, 39.6, 39.5, 39.4, 36.8, 31.4, 30.6, 30.0, 28.6.

**HRMS (ESI^+^)** *m/z* = 1873.9873 [M+2H]^2+^ (calc. for C_181_H_286_N_20_O_63_: 1873.9889 [M+2H]^2+^),
*m/z* = 1895.9755 [M+2Na]^2+^ (calc. for C_181_H_284_N_20_Na_2_O_63_: 1895.9709 [M+2Na]^2+^).

**Figure S1.** HPLC of **1** after reverse phase chromatography purification.

^1^H NMR spectrum of **1**, 500 MHz, 298 K, CD_2_Cl_2_

^13^C NMR spectrum of **1**, 126 MHz, 298 K, CD_2_Cl_2_

**I.4. Evaluation of compound 1 in physiological conditions**

**Stability**

Compound **1** (1.35 mg, 0.7 µmol) was incubated at 37 °C in a solution of 20 mM phosphate buffer at pH 7.2 (0.5 mL) containing 5% of DMSO. Stability in phosphate buffer was monitored by analytical HPLC. HPLC analysis showed no detectable degradation of compound **1** during 24 hours under these conditions.

**Enzymatic hydrolysis**

Enzymatic hydrolysis was carried out with commercial β-glucuronidase from *Escherichia coli* (aqueous glycerol solution, ≥5,000,000 units/g protein, pH 6.8 (biuret)). Compound **1** (1.35 mg, 0.7 µmol) was incubated with the enzyme (133 U) at 37 °C in a solution of 20 mM phosphate buffer at pH 7.2 (0.5 mL) containing 5% of DMSO. Hydrolysis was monitored by analytical HPLC and LC-MS over 2 hours.

**Figure S2.** Stacked HPLC-UV chromatograms: enzymatic hydrolysis of **1** over the time. Retention time: **1**: 8.01 min., **3**: 7.33 min., **4’**: 6.68 min.

**Figure S3.** [A] LC-MS chromatogram TIC (+): enzymatic hydrolysis of **1** after 120 min. [B] LR-MS spectrum of **4’** *m/z* 1021 [M+H]^+^. [C] LR-MS spectrum of **3** *m/z* 1263 [M+2H]2^+^.

**II. BIOLOGICAL SECTION**

**II.1. Cell Culture**

A549 cells (human lung carcinoma CCL-185) and Jurkat (clone E6-1 human acute T-cell leukemia TIB-152) were purchased from the European Collection of Authenticated Cell Cultures (ECACC). Cells were grown in RPMI 1640-GlutaMax supplemented by 10% fetal bovine serum and 100 u/ml Penicillin/Streptomycin in a humidified incubator at 37 °C and 5% CO_2_. Cells were used at early passages (<10).

**II.2. Imaging of cell adhesion**

Cells were grown in full growth media containing 50 µM Ac_4_ManNAz for 3 days. A549 cells were then stained with CellTracker^TM^ green CMFDA for 30 min and treated with 50 µM of marker **2** whereas Jurkat cells were stained with CellTracker^TM^ red CMTPX for 30 min and treated with 50 µM of marker **1** for 30 minutes. After washing with PBS, Jurkat cells were collected and seeded on A549 cells (1:5 A549:Jurkat ratio). After a 10 minutes incubation, non-adherent Jurkat cells were washed whereas A549 and adherent Jurkat cells were fixed prior observation using a FV3000 confocal microscope (Olympus). 3D-analyses were performed using Imaris software.

**II.3. β-glucuronidase-catalyzed cell disassembly assays**

Human bronchial carcinoma A549 cells were seeded onto polylysine D pretreated coverlips in DMEM (Gibco®) supplemented with 10% FBS, 1% penicillin-streptomycin, 1% GlutaMAX and 1% HEPES. Jurkat T cell line was established from the peripheral blood of an acute T cell leukemia patient and cells were seeded into 75 cm² culture flask in RPMI (Gibco®) supplemented with 10% FBS, 1% penicillin-streptomycin, 1% GlutaMAX and 1% HEPES.

For all experiments, A549 and Jurkat T cells were seeded for 24 h (1:5 A549:Jurkat ratio). The medium was replaced for 72 h by fresh medium containing 50 µM of AC_4_ManNAz to functionalize cell surface glycans with azides. The day of the experiment, A549 and Jurkat T cells were incubated with 20 µM of CellTracker^TM^ Green CMFDA or CellTracker^TM^ Red CMTPX, respectively, for 30 min. Besides, Jurkat T and A549 cells were treated with markers **1** and **2** (50 µM; 30 min; 37°C). Thus, A549 cells were stimulated with **2** (A549-[**2**]) whereas Jurkat T cells were incubated with **1** (Jurkat-**[1]**) or **9** (Jurkat-[**9**]). After two washes using fresh medium, A549-[**2**] were co-incubated with Jurkat-[**1**] or Jurkat-[**9**] for 10 min at 37 °C to induce cell-cell interactions. Then, cells were washed twice with fresh medium before ꞵ-glucuronidase stimulation (133 units/mL) for 0, 1, 2, 3, 4, 5 or 6 hours. Following ꞵ-glucuronidase treatment, cells were washed twice with PBS 1X and fixed with 3.7% paraformaldehyde (5 min, RT).

Four to six pictures were randomly captured with the Olympus FLUOVIEW FV3000 confocal microscope for each conditions. Ratios between A549 and Jurkat T cells were measured manually in a blind fashion using imageJ software. These ratios were normalized to non-treated group (ꞵ-Glu-0h) for each condition (ꞵ-Glu-1h, -2h, -3h, -4h, -5h, -6h). Mean and SEM were calculated using Graphpad software from normalized values and final graphs were obtained from three independent experiments.

When A549-[2] were associated with Jurkat-[1] in the presence of β-Glu, the images showed a significant decrease of the Jurkat (red) / A549 (green) ratio along the time (Figure S4). On the other hand, when experiments were conducted with A549-[2] and Jurkat-[9], such an effect was not observed and the Jurkat (red) / A549 (green) ratio remained constant until 6 hours after introduction of β-Glu in the culture medium.


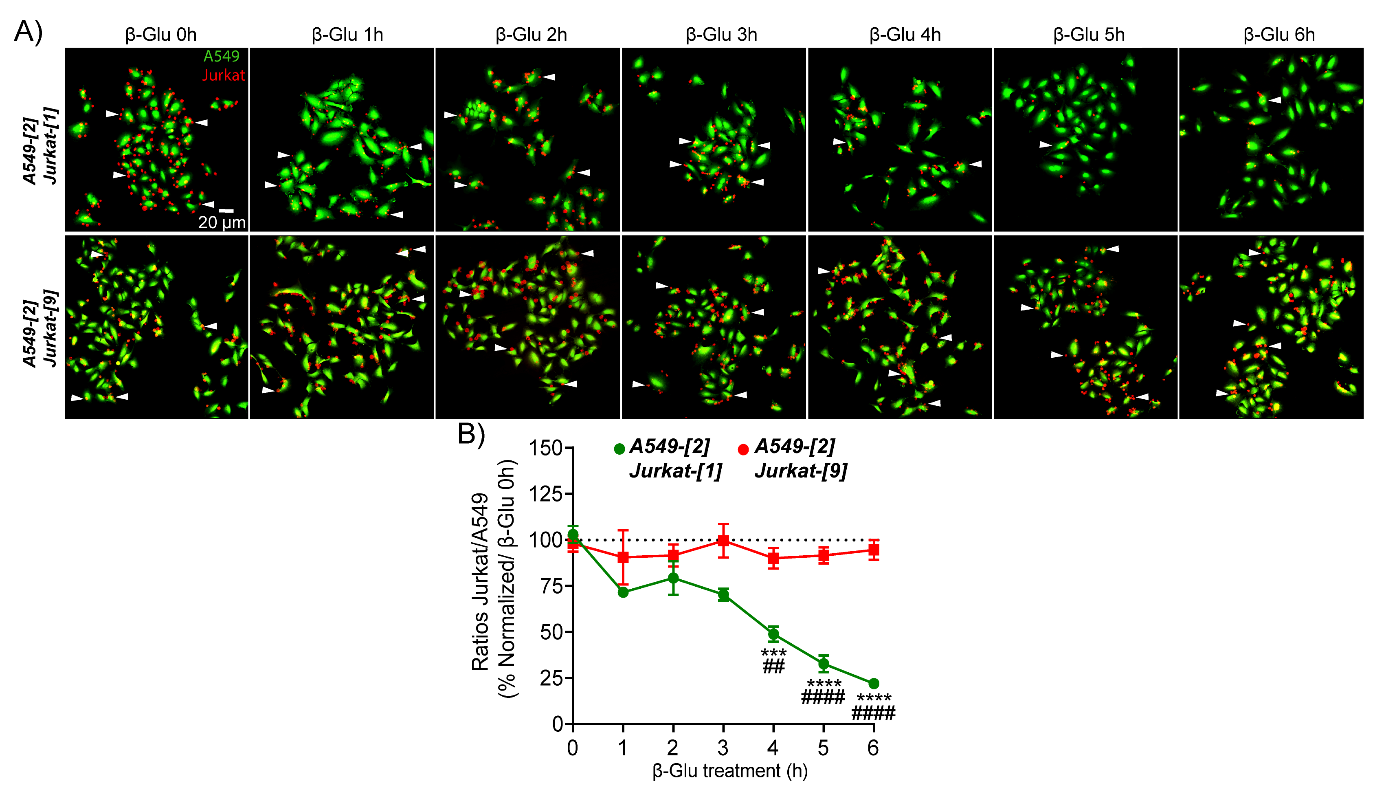


**Figure S4.** A) Confocal microscopy imaging of cellular recognition. A549-[**2**] cancer cells (green) were associated with either Jurkat-[**1**] or Jurkat-[**9**] lymphocytes (red) and β-glucuronidase (133 units/mL) was incubated in the culture media. Pictures show cell association in the absence of β-glucuronidase (β-Glu-0h) and after 1, 2, 3, 4, 5 or 6 hours following addition of the enzyme in the culture media. B) Quantification of the Jurkat-[**1**] / A549-[**2**] and Jurkat-[**9**] / A549-[**2**] ratio along the time after incubation of β-glucuronidase in the culture media. Values represent the mean ± SEM from three independent experiments. Statistical significance was determined by a Two-Way ANOVA test with multiple comparisons followed by Tukey posttest (*** : p **<** 0,001; **** : p **<** 0,0001 = A549-[**2**]/Jurkat-[**1**] + β-Glu-0h vs. A549-[**2**]/Jurkat-[**1**] + β-Glu-4h, -5h, -6h and ## : p **<** 0,01; #### : p **<** 0,0001 = A549-[**2**]/Jurkat-[**1**] vs. A549-[**2**]/Jurkat-[**9**]). β-Glu: β-glucuronidase.

**II.4. Cell proliferation assays**

A549 and Jurkat T cells were seeded, co-incubated and treated as described above to perform immunofluorescence assays with β-glucuronidase. After cells fixation, immunodetection of Ki-67 was performed using anti polyclonal antibody (1 : 200, overnight, 4 °C, Ozyme) and positive signal was revealed with donkey anti-rabbit Alexa Fluor® 647-conjugated secondary polyclonal antibody (1 : 200, 1 h, RT, Thermofisher).

Six pictures were randomly captured with the Olympus FLUOVIEW FV3000 confocal microscope for each condition. Ratios between A549 Ki-67 positive and total A549 were measured manually in a blind fashion using imageJ software. Ratios between Jurkat T Ki-67 positive cells and total Jurkat T cells were calculated in the same way. These ratios were normalized to non-treated group (β-Glu-0h) for each conditions (β-Glu-1h, -2h, -3h, -4h, -5h,
-6h). Mean and SEM were calculated using Graphpad software from normalized values and final graphs were obtained from three independent experiments.


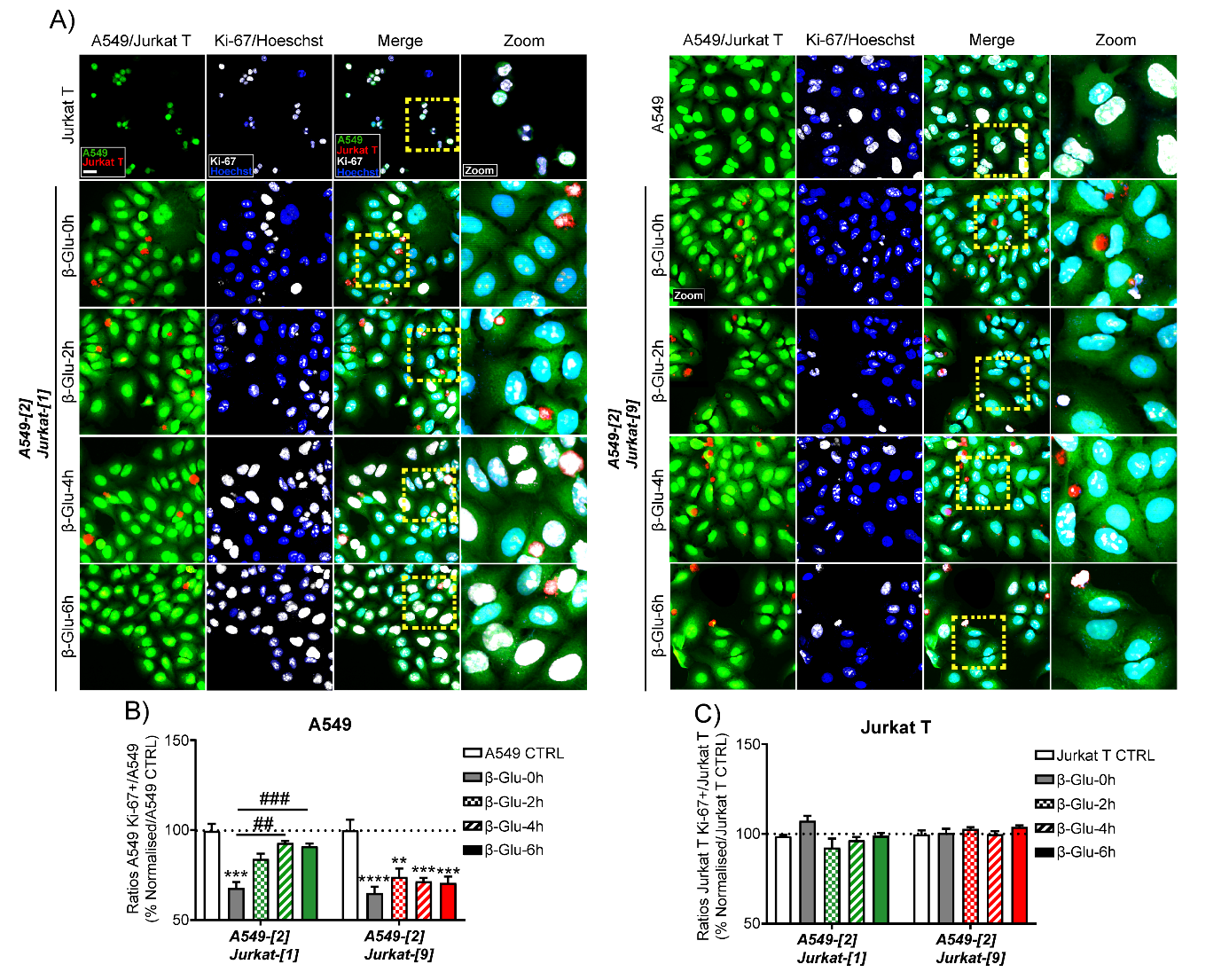


**Figure S5.** **A**) Representative confocal microscopy pictures showing positive Ki-67 signal in A549-[**2**] cancer cells (green) associated with either Jurkat-[**1**] or Jurkat-[**9**] lymphocytes (red). B) Quantification of the positive Ki-67 A549-[**2**] / total A549 ratios along the time after incubation of β-glucuronidase in the culture media for A549-[**2**]/Jurkat-[**1**] and A549-[**2**]/Jurkat-[**9**] association. C) Quantification of the positive Ki-67 Jurkat-[**1**] / total Jurkat or Jurkat-[**9**] / total Jurkat ratios before (β-Glu-0h) or after 2, 4 or 6 hours of β-glucuronidase treatment (β-Glu-2h, -4h and -6h). Values represent the mean ± SEM from three independent experiments. Statistical significance was determined by a Two-Way ANOVA test with multiple comparisons followed by Tukey posttest (** : p **<** 0,01; *** : p **<** 0,001; **** : p **<** 0,0001 = A549 control vs A549-[**2**]/Jurkat-[**1**] or A549-[**2**]/Jurkat-[**9**] + β-Glu-0h, -2h, -4h or -6h and ## : p **<** 0,01; ### : p **<** 0,001 = A549-[**2**]/Jurkat-[**1**] + β-Glu-0h vs. A549-[**2**]/Jurkat-[**1**] + β-Glu-4h or β-Glu-6h). β-Glu: β-glucuronidase.

**II.5. Migration assays**

A549 and Jurkat T cells were seeded in DMEM and RPMI-1640 respectively, supplemented with 10% FBS, 1% penicillin-streptomycin, 1% GlutaMAX and 1% HEPES containing 50 µM Ac_4_ManNAz for 3 days. Then, functionalized A549 were counted and incubated for 16 h in Corning® costar® Transwell® cell culture inserts (Sigma, 3,7.10^4^ cells by Boyden chamber). At this stage, DMEM depleted in FBS was used in the upper and the lower chambers. On the day of the experiment, A549 and Jurkat T cells were treated with 50 µM of markers **1**, **2** or **9** (30 min, 37 °C) respectively. Thus, A549 were incubated with marker **2** (A549-[**2**]) whereas Jurkat T cells were incubated with markers **1** (Jurkat-[**1**]) or **9** (Jurkat-[**9**]). A549 and Jurkat T cells were then co-incubated (1:5 A549:Jurkat ratio) for 10 min at 37 °C and washed twice. Immediately after co-incubation, ꞵ-glucuronidase (β-glucuronidase for *Escherichia Coli*, Sigma-Aldrich ref G8162, 100 μL per well from a stock solution of 133 units/mL) was introduced in half Boyden chambers (6 h, 37 °C). In parallel, the cell proliferation was blocked in all conditions to observe artificial cell-cell interaction effects on A549 migration properties only. Thus, cells were treated with 2,5 µM (1 h, 37 °C) of AKT inhibitor (Protein Inhibitor X, Thermofisher) and MAPK inhibitor (U0126, Thermofisher). All these different treatments were performed using depleted media. Following these different treatments, all Boyden chambers were washed twice with fresh media and cells were incubated for 48 h. Cells in the upper chamber were incubated with depleted DMEM whereas media supplemented with 10% FBS was introduced in the lower chamber to stimulate cancer cells chemotaxis transition. Migrating cells were fixed and colored with crystal violet solution containing 2% ethanol (60s, RT) and pictures were captured with a MacroView MVX10 (Olympus).

For each condition, six fields were randomly chosen to count manually the number of invasive positive crystal violet cells. The values were normalized to non-treated group (A549) for each conditions (A549-[**2**], A549-[**2**]/Jurkat, A549-[**2**]/Jurkat-[**9**], A549-[**2**]/Jurkat-[**1**]) in the presence or in the absence of β-glucuronidase. Mean and SEM were calculated using Graphpad software from normalized values and final graphs were obtained from four to five independent experiments.

**
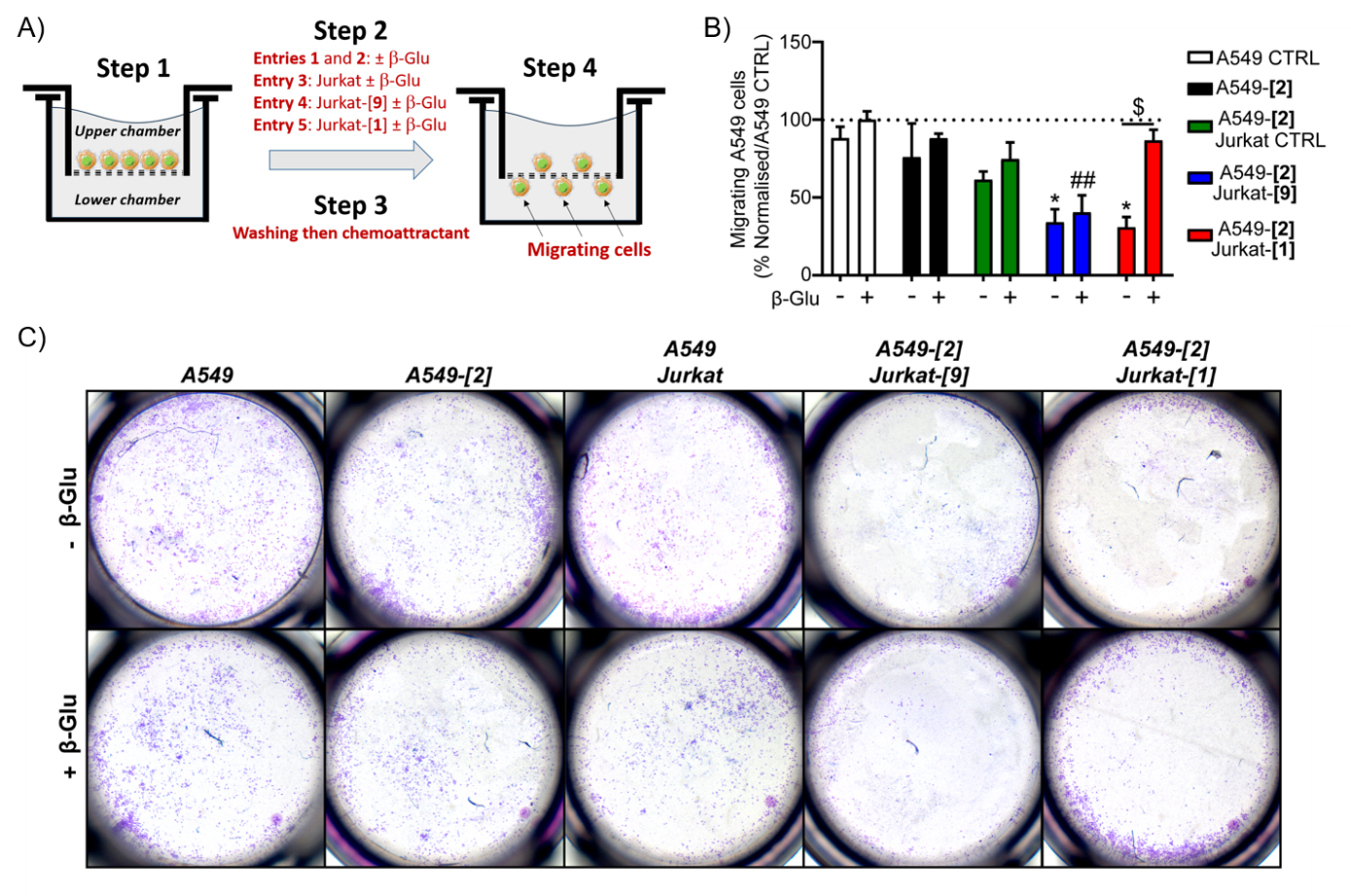
Figure S6.** A) Schematic overview of cell migration assays. Step 1: incubation of A549 or A549-[**2**] in the upper chamber; Step 2: incubation of Jurkat, Jurkat-[**1**] or Jurkat-[**9**] lymphocytes with A549-[**2**] for 10 minutes in the presence or absence of β-Glu; Step 3: washing of the culture medium and introduction of a chemoattractant (10% fetal bovine serum) in the lower chamber; Step 4: migrating cells were fixed and colored with crystal violet prior to be counted. B) Quantification of migrating A549 or A549-[**2**] cancer cells. Values represent the mean ± SEM from four to five independent experiments. Statistical significance was determined by a Two-Way ANOVA test with multiple comparisons followed by Tukey posttest (* : p **<** 0,05 = A549 CTRL *vs* A549-[**2**] + Jurkat-[**9**]/Jurkat-[**1**] without β-Glu; ## : p **<** 0,01 = A549 CTRL *vs* A549-[**2**] + Jurkat-[**9**] with β-Glu and $ : p **<** 0,05 = A549-[**2**]/Jurkat-[**1**] +/- β-Glu. C) Representative pictures showing A549 migrating cells in all conditions. β-Glu: β-glucuronidase.

**II.6. Cell viability assays**

A549 and Jurkat T cells were incubated for 3 days with 50 µM Ac_4_ManNAz in 96 well microplates. On the day of the experiment, A549 and Jurkat T cells were treated or not with 50 µM of markers **1**, **2** or **9** (30 min, 37 °C). Thus, A549 were incubated with marker **2** (A549-[**2**]) and Jurkat T cells were treated with markers **1** (Jurkat-[**1**]) or **9** (A549-[**9**]) and incubated for 24 h at 37 °C. To evaluate the markers effect on cell viability, cell proliferation kit II (Sigma) was used and optic density was measured with a spectrophotometer (490 nm).

The values were normalized to non-treated group (A549 CTRL or Jurkat CTRL) for each condition (A549 vs A549+N_3_, A549-[**2**] or Jurkat vs Jurkat+N_3,_ Jurkat-[**1]**, Jurkat-[**9**]). Mean and SEM were calculated using Graphpad software from normalized values and final graphs were obtained from three independent experiments.


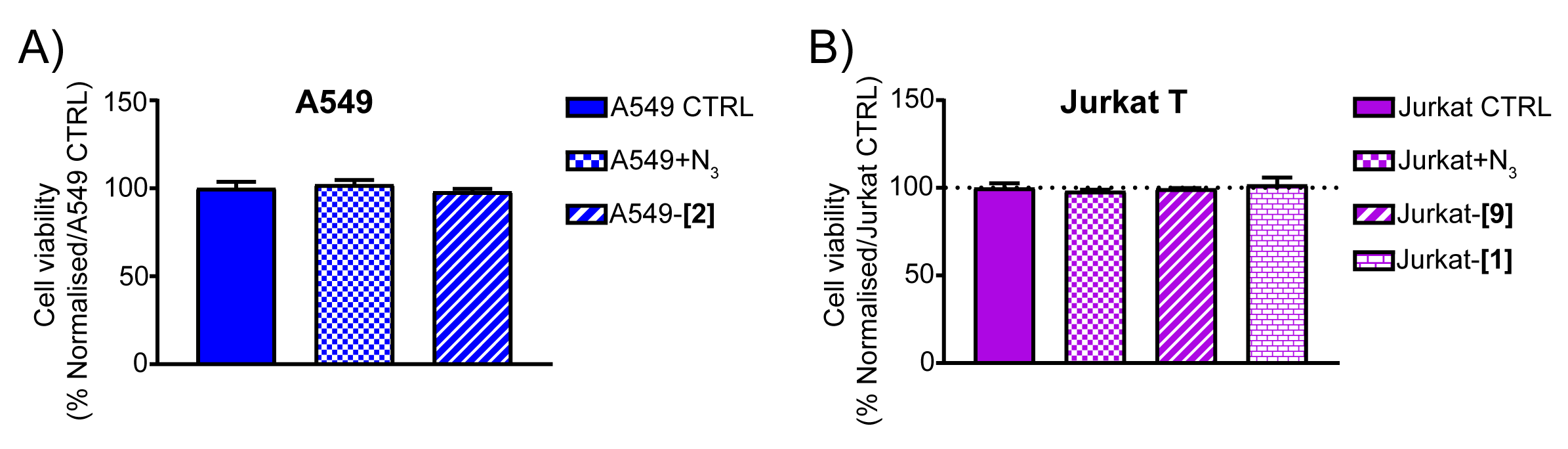


**Figure S7.** A) Viability of A549 cells (A549 CTRL), A549 cells functionalized with azides by metabolic glycoengineering (A549+N_3_) and A549 cells functionalized with the marker 2 (A549-[**2**]). Cell viability was evaluated 24 h after treatments. B) Viability of Jurkat T cells (Jurkat CTRL), Jurkat T functionalized with azides by metabolic glycoengineering (Jurkat+N_3_) and modified Jurkat T cells functionalized with the markers **1** (Jurkat-[**1**]) and **9** (Jurkat-[**9**]). Cell viability of cells was evaluated 24 h after treatments. Values represent the mean ± SEM from three independent experiments. Statistical significance was determined by a Two-Way ANOVA test with multiple comparisons followed by Tukey posttest.

**III. REFERENCES**

1. *Cell-cell interactions via non-covalent click chemistry.* C. Plumet, A. Said Mohamed, T. Vendeuvre, B. Renoux, J. Clarhaut, S. Papot, *Chem. Sci.*, **2021**, *12*, 9017-9021. DOI: 10.1039/d1sc01637g
2. *Targeting the tumour microenvironment with an enzyme-responsive drug delivery system for the efficient therapy of breast and pancreatic cancers.* B. Renoux, F. Raes, T. Legigan, E. Péraudeau, B. Eddhif, P. Poinot, I. Tranoy-Opalinski, J. Alsarraf, O. Koniev, S. Kolodych, S. Lerondel, A. Le Pape, J. Clarhaut and S. Papot. *Chem. Sci.*, **2017**, *8*, 3427-3433. DOI: 10.1039/C7SC00472A, hal-02174996
